# Diversity of Neuromuscular Disorders in Referred Patients to Electro Diagnostic Units of Yazd City

**DOI:** 10.5812/ircmj.4169

**Published:** 2013-03-05

**Authors:** Ahmad Zeinali, Abolghasem Rahimdel, Reza Boostani, Ali Mellat

**Affiliations:** 1 Neurology Department, Shahid Sadoughi Hospital, Yazd University of Medical Science, Yazd, IR Iran; 2 Neurology Department, Qaem Hospital, Mashhad University of Medical Science, Mashhad, IR Iran

**Keywords:** Electrodiagnosis, Neuromuscular Diseases, Carpal Tunnel Syndrome

Dear Editor,

Electrodiagnostic tests are electrical technologies for evaluating the body’s neurophysiology and used as a complementary method for diagnosing the neuromuscular disorders. These tests are commonly used to diagnosis these disorders: Anterior horn cell disease, *Polyneuropathy,* *Mononeuropathy*, *Plexopathy* *, Radiculopathy*, *Polyradiculoneuropathy*, *Neromuscular* junction and Muscular disorders (1, 2). Because the prevalence and demographic features of disorders referred to electrodiagnosis departments are undetermined, this study was performed. People enrolled the study were patients whom were referred to electrodiagnosis units in Yazd from June 2009 to June 2011.Studied variables were: age, sex and test results included: normal, carpal tunnel syndrome (CTS), cervical and lumbar radiculopathy (Cer. Rad, Lum. Rad.), trauma, peripheral neuropathy (P.N.), nerve entrapment (N.E.), myopathy, motor neuron disease (M.N.D.), myotonia, neuro-muscular junction (N.M.J) disorders (myasthenia). In two years consecutive study, EMG-NCV findings of 2933 patients were analyzed. 1651 patients were females and 1282 patients were males which female gender was more significantly (P < 0.001). Patients’ mean age was 43.2 ± 16.4 which 1805 patients were more than 40 and 1128 were under 40 years old (P < 0.001). CTS, lumbar and cervical radiculopathy and peripheral neuropathy were more in patients over 40 years old and trauma, motor neuron disease, myotonia and myasthenia were more in patients under 40 years old significantly(P < 0.001). Numerous studies emphasized the more prevalence of CTS in patients over 40 years because of osteoarthritis and diabetes mellitus (3-5). More normal reports in patients over than 40 yeras old are may be due to rheumatologic disorders, neuromuscular pain with articular, tendonal or muscular origin in which may lead to false diagnosis and refer normal patients for EMG-NCV (6-8). Normal variable and CTS were more in females and cervical radiculopathy, trauma, nerve entrapment and myotonia were more in males significantly (P < 0.001). Several studies indicate more prevalence of CTS 3 to 7 times in females (3-5). In our study this ratio was 6.5/1. In this study cervical radiculopathy was more prevalent in men like other studies (P = 0.024) 9. 32.3% of reports were normal which may be due to high prevalence of soft tissue disorders, muscular pain around shoulder and pelvic griddles such as bursitis, tendonitis and myofacial pain which may be misdiagnosed as radicular pain or other peripheral diseases (6-8). So obtaining precise history and performing complete general and neurologic examination could lead to decrement the referring rate of patients with diseases other than motor unit involvement lead to lowering cost of EMG-NCV for health and treatment system (Table 1).

**Table 1:** Prevalence Distribution of Findings Based on Age and Sex

	Findings										
	Normal	CTS	Lum. Rad	Cer. Rad	Trauma	P. N	N. E	MND	Myopathy	Myotonia	N.M.J
**Age, y, No.**											
< 20	83	9	9	2	60	30	18	16	14	6	2
20-29	177	30	13	6	89	18	29	5	6	7	3
30-39	233	117	41	11	72	16	13	5	11	2	2
40-49, No.	260	228	89	24	70	31	21	10	12	2	1
50-59.	109	216	73	29	22	47	19	3	11	3	0
> 60	85	129	119	31	21	85	17	7	3	1	0
**P value**	0.205	< 0.001	< 0.001	< 0.001	< 0.001	< 0.001	0.782	0.007	0.04	0.002	0.007
**Sex, No.**											
Male	422	96	170	60	261	125	70	25	24	16	4
Female	525	633	174	43	73	102	47	21	33	5	4
**P value**	< 0.001	< 0.001	0.829	0.024	< 0.001	0.127	0.026	0.555	0.233	0.016	1
**Total, No. (%)**	8 (0.3)	21 (0.7)	57 (1.9)	46 (1.6)	117 (4)	227 (7.7)	334 (11.4)	103 (3.5)	344 (11.7)	729 (24.9)	947 (32.3)
